# A proposed experimental diagnosing of specular Andreev reflection using the spin orbit interaction

**DOI:** 10.1038/srep29279

**Published:** 2016-07-08

**Authors:** Yanling Yang, Bing Zhao, Ziyu Zhang, Chunxu Bai, Xiaoguang Xu, Yong Jiang

**Affiliations:** 1 School of Materials Science and Engineering, University of Science and Technology Beijing, Beijing 100083, People’s Republic of China; 2 School of Physics, Anyang Normal University, Anyang 455000, People’s Republic of China

## Abstract

Based on the Dirac-Bogoliubov-de Gennes equation, we theoretically investigate the chirality-resolved transport properties through a superconducting heterojunction in the presence of both the Rashba spin orbit interaction (RSOI) and the Dresselhaus spin orbit interaction (DSOI). Our results show that, if only the RSOI is present, the chirality-resolved Andreev tunneling conductance can be enhanced in the superconducting gap, while it always shows a suppression effect for the case of the DSOI alone. In contrast to the similar dependence of the specular Andreev zero bias tunneling conductance on the SOI, the retro-Andreev zero bias tunneling conductance exhibit the distinct dependence on the RSOI and the DSOI. Moreover, the zero-bias tunneling conductances for the retro-Andreev reflection (RAR) and the specular Andreev reflection (SAR) also show a qualitative difference with respect to the barrier parameters. When the RSOI and the DSOI are finite, three orders of magnitude enhancement of specular Andreev tunneling conductance is revealed. Furthermore, by analyzing the balanced SOI case, we find that the RAR is in favor of a parabolic dispersion, but a linear dispersion is highly desired for the SAR. These results shed light on the diagnosing of the SAR in graphene when subjected to both kinds of SOI.

Over the last 12 years, since it was first isolated by Andre Geim and Kostya Novoselov in 2004, enormous efforts have been devoted to understand and utilize the ‘star material’ –graphene^1^,^2^,^3^. As an ideal two-dimensional (2D) platform for mimicing Dirac fermions in quantum electrodynamics, graphene displays various oddball features which have been revealed and experimentally confirmed, such as, half-integer quantum Hall effects^4^, sub-Poissonian shot noise^5^, valley-dependent Brewster angles^6^, and Klein tunneling^7^.

It is also interesting that an exotic and robust new phase of condensate matter-topological insulator (first termed as quantum spin Hall insulator) might occur naturally as the ground state of the single plane of graphene^8^. Recent advances in materials engineering and fabrication give a series of topological structures beyond 2D graphene, including quantum wells structure^9^,^10^ and 3D crystalline insulator^11^. Unfortunately, the symmetry-protected topological state has not been observed experimentally in graphene up until now, though it was firstly reported in their pioneering work by Kane and Mele. The key obstacle to the experimental discovery of topological state is the small energy gap in graphene. Indeed, the strength of the intrinsic Desselhaus spin orbit interaction (DSOI) in graphene estimated by many studies is very small, about 0.05–0.0011 *meV*^12^,^13^,^14^,^15^. However, realizing the new topological state in graphene may be possibile with strong DSOI made from an interface with a tungsten disulfide substrate very recently^16^. Numerical calculations suggest two to three orders of magnitude enhancement of the DSOI (up to 17 *meV*)^16^. Besides the DSOI induced by bulk inversion asymmetry, which can be tuned by exploiting interfacial interactions^17^, there is another type of spin orbit interaction (SOI) induced by structure inversion asymmetry, the Rashba spin orbit interaction (RSOI), which can be tuned by the external gate voltages^12^,^13^,^14^,^15^, adatoms^18^, and substrate emerging^19^. Essentially, it has been reported experimentally that the RSOI strength can reach values up to 200 *meV* at room temperature^19^. Since the SOI strengths are sufficiently notable and can be tuned by external elements, the observation of the topological states in graphene and the exploitation of the SOI-resolved graphene-based devices appear particularly promising.

Along with the metallic, semiconducting, and topological insulating states, superconducting state is one of the major and the amazing states in graphene. Although it is not superconducting, pristine graphene has also attracted considerable attention due to the unique chance to bridge the gap between relativity and superconductivity in experiment and the possibility of acquiring superconductivity via the hybridization with ambient environment^1^,^2^,^3^. Experimentally, the observation of Josephson supercurrent in graphene Josephson junction suggests that superconductivity can be induced in graphene in the presence of a conventional superconducting lead by means of the proximity effect^20^,^21^,^22^,^23^,^24^. On the other hand, considerable experimental efforts as well as many theoretical proposals are devoted to hunting for the superconducting graphene by doping^25^,^26^,^27^. However, until recently, Chapman *et al*. experimentally show that graphene crystals decorated with Ca exhibit robust superconductivity with a transition temperature 12 *K*^28^. This fundamental property would open a new pathway for exploring the exotic physics and the graphene-based superconducting quantum devices.

The most important prediction of the superconducting heterojunction, which is a combine of a superconductor and a non-superconducting state material, is the two particles transport process at energies less than the superconducting energy gap–Andreev reflection (AR)^29^. Usually the AR in conventional materials is retro-reflection (the reflected hole travels along the same path of the incident electron). However, due to a Dirac-like energy dispersion in graphene, a specular Andreev reflection (SAR) (the reflected hole travels along the specular path of the incident electron), one of the peculiar hallmarks of the graphene-based superconducting physics, occurs^30^,^31^,^32^,^33^. Following the pioneering work in graphene^30^, Lv *et al*. and Majidi *et al*. reported that the specular Andreev reflection can be also found in a conventional semiconductor^34^ and a thin film topological insulator^35^, respectively. In graphene, under completely ideal conditions, this unusual SAR should be experimentally detectable at the Fermi energy reducing to the charge neutrality point. Recently, Zhai *et al*. have investigated the modulation of SAR by the RSOI both in graphene monolayer and in graphene bilayer^36^. It is suggested that the topologically nontrivial changes of Berry phase by the RSOI result in a significant reduction of SAR in graphene monolayer and an obvious enhancement in graphene bilayer. In practice many efforts have been made for searching for the SAR. Nonetheless, such an experiment is extremely difficult and hindered by the inevitable presence of the charge carrier scattering and the strong potential fluctuations^23^,^37^. To overcome the obstacles, the suspended^38^ and hBN supported graphene samples^39^ have been employed to dramatically reduce these strong potential fluctuations^40^. Meanwhile, the dry-vdW pick-up technique and a current annealing method^41^,^42^ are adopted to realize ultra-clean, atomically sharp, and highly transparent Ohmic interfaces between graphene and superconductor junctions. More recently, a considerable experimental progress has been steadily made in hunting the SAR where an unprecedentedly clean bilayer graphene-based superconducting heterojunction has been developed and a key point for the transition between the usual retro-Andreev reflection (RAR) and the special SAR has been revealed^43^. With the experimental technology advancement, the surge of interest in testing the SAR in single-layer graphene would be realized in the coming years.

However, there is no report on the combined effect of the DSOI on the AR in graphene-based superconducting heterojunction. Since both the SOI strengths are sufficiently notable and also tunable, here we investigate the spin-resolved transport properties through graphene-based superconducting heterojunction in the presence of the SOI. The effect of the SOI on the process of AR and the tunneling conductance is extensively investigated. The numerical results indicate that the AR (the RAR and the SAR) not only can be greatly modulated by the strength of the SOI, but also exhibits a strikingly different dependence on the SOI (DSOI and RSOI). Moreover, the sub-gap tunneling conductance for the SAR case, in sharp contrast to its counterpart in conventional RAR case where it always increases with increasing incident energy, becomes non-monotonic and reaches a maximum at a certain finite incident energy. The emergence of those qualitative differences resulted from the acquisition of the AR hole localized in which band (conduction or valance) may give birth to a new way to diagnose the SAR in the actual experiments.

## Model and Basic Formalism

**Figure 1:**
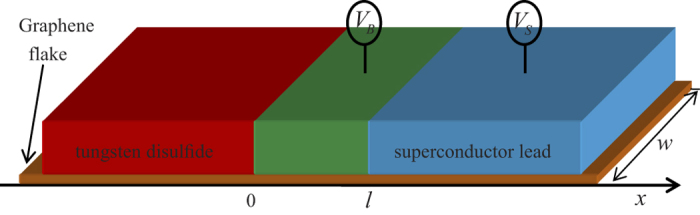
Sketch of the superconducting *heterojunction*. *l* is the length of the central barrier lead. *V*_*B*_ and *V*_*S*_ are the voltage applied to the barrier and superconducting lead, respectively. The red lead represents a tungsten disulfide lead which causes a notable strength of the DSOI in graphene. The light blue lead represents a conventional s-wave superconducting lead which induces the superconductivity in graphene via the proximity effect.

We consider a spin-resolved electron passing through a graphene-based region with a potential barrier which is sandwiched by graphene-based superconducting lead and normal metal lead in which the SOI is present. The geometry sketch of the heterojunction is shown in Fig. 1. The growth direction is taken as the *x* axis. The normal metal lead with the SOI extends from *x* = −∞ to *x* = 0, the barrier region, modeled by a barrier potential *V*_0_, extends from *x* = 0 to *x* = *l*, and the superconducting region occupies *x* > *l*. Here the RSOI in the normal metal lead can be realized by either growing graphene on a Ni surface or growing graphene on the Surface of SiC^19^,^44^. Meanwhile the DSOI can be induced in the region by exploiting interfacial interactions (with a tungsten disulfide flake)^16^,^17^. Such a local barrier can be implemented by either using the electric field effect or local chemical doping^1^,^7^,^45^. The region *x* > *l* is to be kept close to a conventional superconducting lead so that superconductivity is induced in this region via the proximity effect^20^,^21^,^22^,^23^,^24^. In the rest of this study, we focus on the case where the width (along y direction) of the graphene strip, *w*, is much larger than *l* (*l* << *w*). In this manner, details of the microscopic description of the strip edges become irrelevant, and can be realistically created in experiments^1^,^7^,^45^.

Throughout this study we consider a defect-free graphene sheet in the xy-plane. To grasp the essential physics, we shall restrict ourselves to a single particle picture and neglect electron-electron interaction effect. The low energy excitation quasiparticles propagation in the present superconducting heterojunction can be described by the following Dirac-Bogoliubov–de Gennes (DBdG) equation^30^,^31^,^32^,^33^


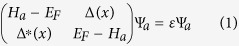


Here, 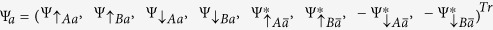 is the 8 component wave functions for the electron and hole spinors, where Tr represents transpose. The arrow index (↑, ↓) stands for real spin, the index *a* denotes *K* or *K*′ for electrons or holes near *K* and *K*′ points, 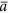 takes values *K*′(*K*) for *a* = *K*(*K*′), *A* and *B* denote the two inequivalent sites in the hexagonal lattice of graphene, *E*_*F*_ denotes the Fermi energy, Δ(*x*) is superconducting pair potential which can be modeled as Δ(*x*) = Δ_0_*e*^*iφ*^*θ*(*x*), where Δ_0_ and *ϕ* are the amplitude and the phase of the induced superconducting order parameters, respectively, and *θ*(*x*) is the Heaviside step function, and the valley-resolved Hamiltonian *H*_*a*_ is given by


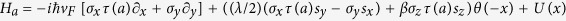


where *v*_*F*_ ≈ 10^6^ *ms*^−1^ is the Fermi velocity, the Pauli matrice 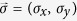 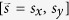 describes the sublattice [the spin] degree of freedom, *τ*(*a*) is the valley index (*τ*(*a*) is 1(−1) for *a* = *K*(*K*′)), and the parameters *λ* and *β* represent the strength of the RSOI and of the DSOI, respectively, the last term *U*(*x*) describes the distribution of the electrostatic potential across the heterojunction. The electrostatic potential may be adjusted independently by a gate voltage or by doping. Moreover we also assume that the potential vary is sharp on the scale of the Fermi wavelength in each of the interfaces. Since the zero of potential is arbitrary, we thus investigate the scattering through an abrupt potential defined by


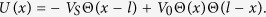


In the following we set *ħ* = *v*_*F*_ = 1. In this paper, besides the spintronics relevant regime *λ* >> *β*, we will consider the regime *β* >> *λ* because the topological insulate phase occurs in this regime. The other intriguing scenario is present at the balanced case (*λ* = 2*β*) where the spectrum is gapless and hosts a Dirac cone.

For a chirality-resolved electron incident on the junctions from the left lead with energy *ε*, transverse momentum *q*, and chirality *γ*, the wave functions in the three regions, taking into account both normal reflection and Andreev reflection processes, can be written as


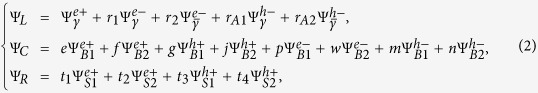


where *r*_1_ and *r*_2_ are the amplitudes of normal reflections, respectively, *r*_*A*1_ and *r*_*A*2_ are the amplitudes of Andreev reflections, respectively, *e*, *f*, *g*, *j*, *p*, *w*, *m*, and *n* are the amplitudes of electron and hole in the central barrier region, and *t*_1_, *t*_2_, *t*_3_, and *t*_4_ are the amplitudes of electron and hole quasiparticles in the right superconducting region. *γ* and 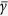 denote the chirality, and 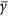 is − for *γ* is +. ± denotes the wave functions traveling along the ±*x* direction in the heterojunction. The specific form of the wave functions can be obtained by solving the Eq. (1), and they can be clearly expressed in the different regions. Note that we also assume an ideal interface with translational invariance along the y direction. Thus the momentum along the y axis, *q*, is a good quantum number and factor *e*^*iqy*^ can be omitted accordingly.

In the left normal metal lead, the wave functions are given by


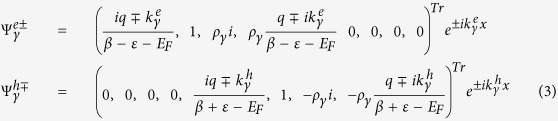


where 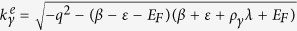 and 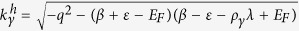 are the momentum along the *x* axis for the electron and hole quasiparticle, respectively, *ρ*_*γ*_ = 1(−1) for *γ* is −(+). Note that those wavevector might get an imaginary number when the transverse momentum *q* is beyond a critical value. However, those evanescent solutions in the calculations should be considered in the scattering process to ensure the appropriate current conservation.

In the central barrier region, one can similarly obtain


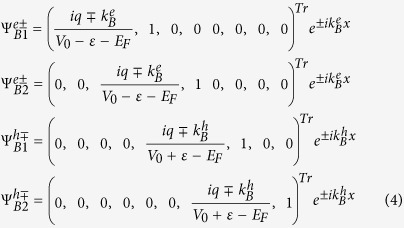


where 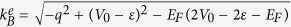 and 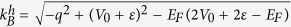 are the momentum along the *x* axis for the electron and hole quasiparticle, respectively.

In the right superconducting region, though the DBdG quasiparticles are mixtures of electron and hole quasiparticle, we can show the wavefunctions in the similar manner as


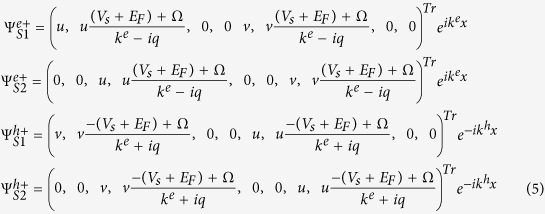


where 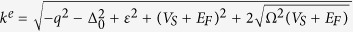 and 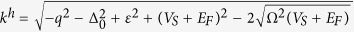 are the momentum along the *x* axis for the electron and hole quasiparticle in the superconducting lead, respectively, the coherence factors are given by 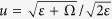, 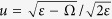, and 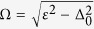.

All the amplitudes in Eq. (2) can be determined by applying wavefunction continuity at the interfaces. These boundary conditions are given by


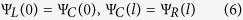


Using the boundary conditions Eq. (6), the amplitudes *r*_1_, *r*_2_, *r*_*A*1_, and *r*_*A*2_ in Eq. (2) for the present superconducting heterojunction can be obtained straightly. Once scattering amplitudes are obtained, the zero temperature tunneling conductance for our system can be calculated by means of the extended Blonder-Tinkham-Klapwijk formula^46^,^47^,^48^,


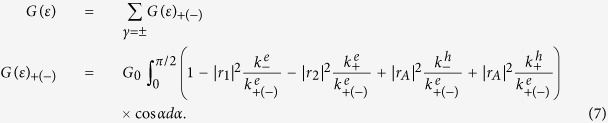


where *G*_0_ = 2*e*^2^*N*(*ε*)/*h* is half of the ballistic conductance of metallic graphene (the number 2 denotes a two-fold valley degeneracy), *α* is the quasiparticle’s incident angle, and *N*(*ε*) = (*E*_*F*_ + *ε*)*w*/(*πħv*_*F*_) denotes the number of available channels for a graphene sample of width *w*. Using Eq. (7), the tunneling conductance for the present superconducting heterojunction can be obtained easily by the numerical calculations.

## Results and Discussion

### Excitation spectrum

**Figure 2:**
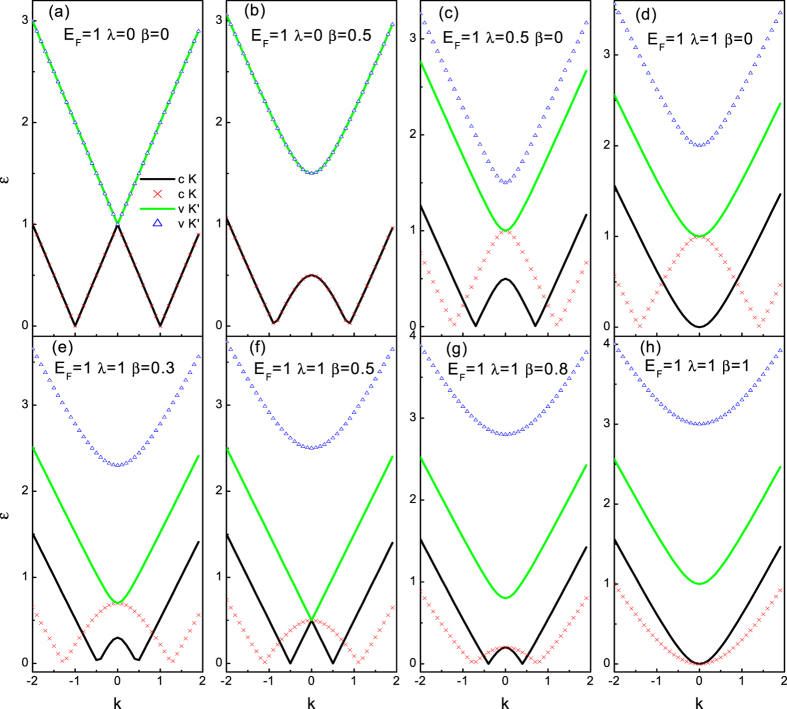
Spectrum of the DBdG equation with DSOI and RSOI as a function of *k*_*x*_ for *q* = 0, calculated with Δ(*x*) = *U*(*x*) = 0 (*x* < 0) for the Fermi energy *E*_*F*_ = 100Δ_0_. Black lines and × lines indicate quasiparticle excitations with chirality + and − in the conduction bands, while green lines and △ lines indicate quasiparticle excitations with chirality − and + in the valence bands. The quasiparticle excitation type (electron/hole) is determined by the derivative of the dispersion relation. Panel (**a**): *λ* = *β* = 0. Panel (**b**): *λ* = 0 and *β* = 0.5. Panel (**c**): *λ* = 0.5 and *β* = 0. Panel (**d**): *λ* = 1 and *β* = 0. Panel (**e**): *λ* = 1 and *β* = 0.3. Panel (**f**): *λ* = 1 and *β* = 0.5. Panel (**g**): *λ* = 1 and *β* = 0.8. Panel (**h**): *λ* = 1 and *β* = 1.

**Figure 3:**
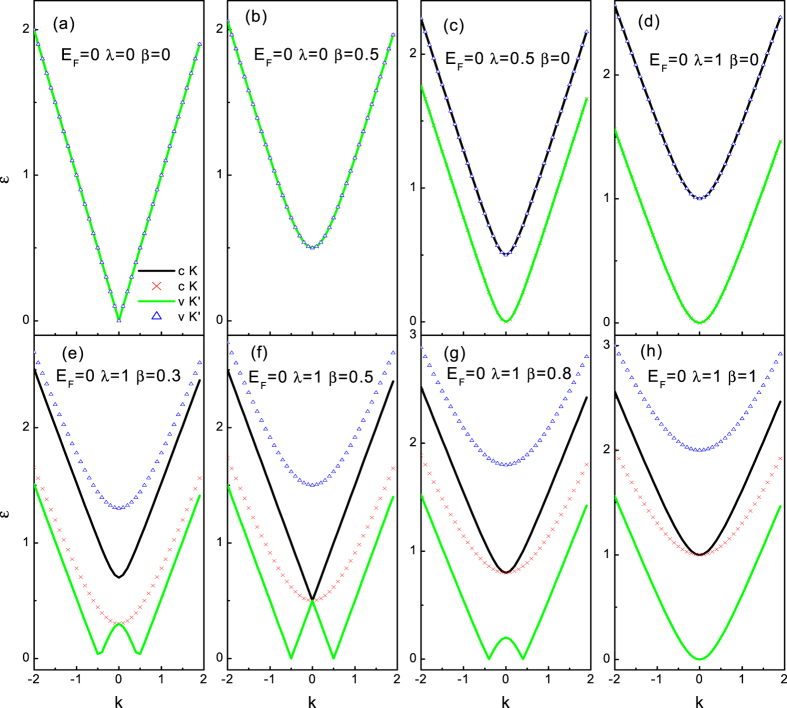
Spectrum of the DBdG equation with the DSOI and the RSOI as a function of *k*_*x*_ for *q* = 0 and the Fermi energy *E*_*F*_ = 0. The lines and parameters are similar to the Fig. 2.

We start our analysis from the excitation spectrum of the DBdG equation. The excitation energy *ε* is measured relative to the Fermi energy *E*_*F*_ and can be given as 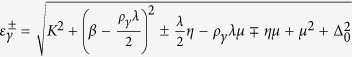 with 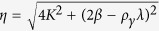, *μ* = *E*_*F*_ − *U*(*x*), and 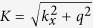. Note that the *x* component of momentum *k*_*x*_ in the left, central, and right lead corresponds to 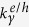, 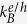, *k*^*e*/*h*^, respectively. The excitation spectrum 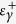 and 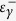 originate from the conduction band and the valence band, respectively. It is well-known that the SAR occurs when the reflected hole travels in the valence band below the neutrality point, while the RAR corresponds to a conduction band hole. That is to say, SAR dominates in weakly doped normal metal graphene lead (the Fermi energy *E*_*F*_ stays around the neutrality point), while in the case of heavily doped normal metal graphene lead (the Fermi energy *E*_*F*_ stays far away from the neutrality point), the RAR is dominant. Therefore, to compare the effect of the SOI on SAR with that on RAR, we will investigate the two limiting regimes *E*_*F*_ = 100Δ_0_ and *E*_*F*_ = 0 separately. The qualitative differences in the excitation spectrum as well as in the scatting process at the two limiting cases are expected. The dispersion relations about excitation energy *ε* are shown in Figs 2 and 3 for the normal metal region (where Δ(*x*) = *U*(*x*) with *x* < 0).

First we consider the case of *E*_*F*_ = 100Δ_0_, as shown in Fig. 2. In the absence of SOI effect, clearly seen in Fig. 2(a), we find the well known results of ref. 30. For an electron excitation (above the Fermi level), the reflected hole state (below the Fermi level) also lies in the conduction band. Note that both the electron and hole excitation bands are remains particle-hole symmetry and spin degeneracy in this case. In the absence of RSOI, the DSOI opens a gap at *k*_*x*_ = 0, each one still remains particle-hole symmetric and a two-fold degeneracy with respect to spin degrees of freedom. This is in contrast to the energy band-split produced by the DSOI in the conventional semiconductor where each band state becomes completely spin-resolved^49^. It is also important to note that the band (below the Fermi energy) is suppressed by the DSOI, which may eventually close the band. Therefore the RAR (the reflected hole travels in conduction band) is shut down when the DSOI exceeds a certain threshold (*β* = *E*_*F*_). Moreover, unlike the case of absence of the SOI (the well known graphene linear energy dispersion), the excitation bands have parabolic dispersion near the Fermi energy. In the absence of the DSOI, though it does not give rise to a gap (one pair of the dispersion branches is gaped and the other pair of does not exhibit a gap), but the RSOI does lift the spin degeneracy, as seen in Fig. 2(c,d). In this case, the chirality + band (below the Fermi energy) is suppressed by the RSOI, and eventually disappeared, which leads to the RAR forbid in the channel. It is important to remark that the RSOI in graphene is strikingly different from that in the conventional semiconductor where the RSOI just induce a spin-resolved horizontal splitting^47^. However, because of the valley freedom involved the chirality-resolved splitting in graphene does not break the time-reversal symmetry. As of both the DSOI and the RSOI are finite, the excitation spectrum lifts particle-hole symmetry and exhibits a richer structure, as illustrated in bottom panel (e), (f), (g), (h). With the increase of the DSOI, the disappeared chirality + band (below the Fermi energy) appears again and grows up. The balanced DSOI and RSOI case (*λ* = 2*β*) is rather special, as shown in Fig. 2(f). First, the chirality + band below the Fermi energy reachs its maximum. Second, one pair of linearly dispersing bands are recovered while the other pair of chirality-split bands are still parabolic. In this manner, the amazing thing is that we have the unique possibility to test and compare AR occurs between the bands with different dispersion curves which cannot be realized in a pristine graphene. When the DSOI dominates (*β* > *λ*), both the chirality-resolved bands (below the Fermi energy) die away with the increase of the DSOI. That is to say, the Mexican-hat-like shape conduct bands transform into the parabolic bands for the DSOI beyond a certain value (*β* = *λ*). As a result, two evanescent RAR waves appear and yield unit modular reflection amplitude, thereby indicating a perfect reflection.

The energy dispersion for *E*_*F*_ = 0 as a function of *k*_*x*_ at fixed *q* = 0 is illustrated in Fig. 3 for several values of the DSOI and the RSOI. Besides the similarities to the case of *E*_*F*_ = 100Δ_0_, we find that the excitation spectrum of quasiparticle exhibits several unusual features. First, the chirality-resolved conduction and valence bands coincide with each other (in the case of without or with only one kind of the SOI) which means that the SAR happens (an electron in the conduction band is converted into a hole in the valence band). Second, when both the SOI are finite-the situation is shown in bottom panel ((e)–(h))-the excitation spectrum becomes more intriguing. With the increase of the DSOI, the chirality + valance band deforms into a Mexican-hat-like shape in the lowest energy (around *k*_*x*_ = 0). When the DSOI is not too large, the Mexican hat feature remains, but disappears as the DSOI is increased further (beyond *β* = *λ*), see Fig. 3(e–h). The center height of this Mexican hat is equal to 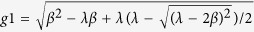. In the energy interval [0, *g*1], the electron-hole conversion occurs in the different valleys of the same valence band, i.e., a RAR thus occurs. Above the energy regime, the electron-hole conversion happens with an electron in the conduction band and a hole in the valence band, i.e., a SAR. Note that the behavior in the energy interval [0, *g*1] is similar to that described in the above, but the different thing is that the RAR takes place in the valence band here (while it occurs completely in the conduct band above).

### Tunneling conductance

#### Only one kind of the SOI

The tunneling conductance for the superconducting heterojunction as a function of the incident energy *ε* for the different *λ* and *β* has been calculated by equation (7). In this section we focus on the case where only one kind of the SOI (DSOI or RSOI) is present. Notice that in the superconducting region, the mean-field requirement is satisfied as long as Δ_0_ << *E*_*F*_ + *V*_*S*_, thus, in principle, for a large *V*_*S*_ one can reach the regimes where Δ_0_ >> *E*_*F*_ (*E*_*F*_ = 0). The magnitude of the Fermi energy in the normal region is not constrained, and we will have the chance to compare the two regimes *E*_*F*_ = 100Δ_0_ and *E*_*F*_ = 0. Therefore, the parameter *V*_*S*_ used in the calculation are *V*_*S*_ = 100*E*_*F*_ with *E*_*F*_ = 100Δ_0_ and *V*_*S*_ = 100Δ_0_ with *E*_*F*_ = 0. First we assume a perfect junction interface, i.e., *l* = 0 and *V*_0_ = 0.

**Figure 4:**
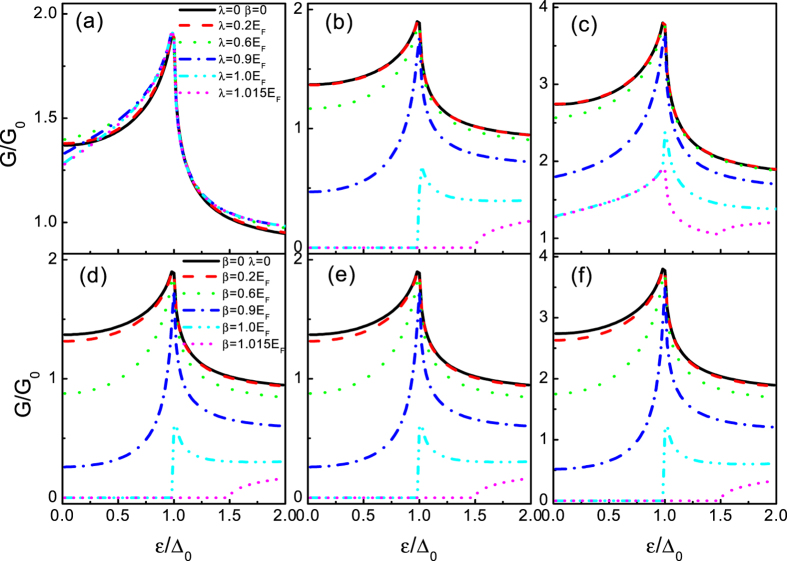
The tunneling conductance for the superconducting heterojunction as a function of the incident energy *ε* for the different *λ* (**a–c**) and *β* (**d–f**). From the left panel to the right panel, *G*′ = *G*_−_, *G*_+_, *G*. The parameters used in the calculation are *l* = 0, *V*_0_ = 0, *V*_*S*_ = 100*E*_*F*_, and *E*_*F*_ = 100Δ_0_. The other parameters are shown in the figure.

Figure 4(a–c) correspond to the tunneling conductance for *E*_*F*_ = 100Δ_0_ when only the RSOI exists, while Fig. 4(d–f) correspond to the case where only the DSOI exists. When only the RSOI exists (Fig. 4(a–c)), as the strength of the RSOI increases, it is clear to see that the tunneling conductances for chirality + and − exhibit different features. The distinction attributes to the excitation spectrum revealed above. Specifically, the chirality − conduct band and its chirality + valence band touch at the Dirac point, as seen in Fig. 2(c,d). In contrast to the case of the chirality − conduct band, the chirality + conduct band and its chirality − valence band yield a gap 2*λ* by the RSOI. At the large enough RSOI, namely *λ* > *E*_*F*_, there is one propagating (chirality −) and one evanescent (chirality +) transmission waves which results in a finite and zero tunneling conductance, respectively. Things become more intriguing when we only consider the case of the chirality − conduct band incident. In Fig. 4(a), the variation of the tunneling conductance with *ε*/Δ_0_ is plotted for several values of *λ*. In a conventional two-dimensional electron gas/insulator/superconductor junction with the RSOI, the tunneling conductance monotonically decreases with increasing the RSOI (ref. 44), because of the reduction of the Andreev reflection. In physics, the RSOI lifts the spin degeneracy that induces a wave vector splitting between the incident electron and the reflected hole, which results in the suppression of the RAR. However, in graphene, the Fermi surface is chirality-resolved (also spin-resolved), and splits into two due to the RSOI, which makes the tunneling conductance features more interesting. As clearly seen from the Fig. 4(a), in the presence of the RSOI (the strength *λ* almost up to 0.7*E*_*F*_), the RAR is enhanced and therefore the subgap tunneling conductance in the entire superconducting gap region 0 ≤ *ε* ≤ Δ_0_ while it is almost unchanged near the gap edge. The enhanced tunneling conductance can even have a maximum at *ε* = 0 at certain values of the RSOI *λ* ≈ 0.6*E*_*F*_. When the RSOI is beyond the threshold *λ* ≈ 0.7*E*_*F*_, the subgap tunneling conductance is enhanced in the region *ε*′ ≤ *ε* ≤ Δ_0_, while it is suppressed in the energy regime 0 ≤ *ε* < *ε*′. These features are quite different from those observed in the conventional junction where the monotonical reduction stems from the energy split by the RSOI^47^.

When the DSOI is alone, in turn, see Fig. 4(d–f), the tunneling conductances for the chirality + and − coincide with each other due to the spin degeneracy (the DSOI does not lift the spin degeneracy). Moreover, we can find that the tunneling conductances (*G*_+_ and *G*_−_) for the case of the DSOI alone are the same as *G*_+_ for the case of the RSOI alone. This can be intuitively elucidated by the similar excitation spectrum of those cases, as shown in Fig. 2(b,c). From the above analysis, we can draw a conclusion that the DSOI opens a gap in the excitation spectrum (both chirality), thus it has a great impact on the transport properties of quasiparticles in the system, as compared to the RSOI (only one chirality band opens a gap).

**Figure 5:**
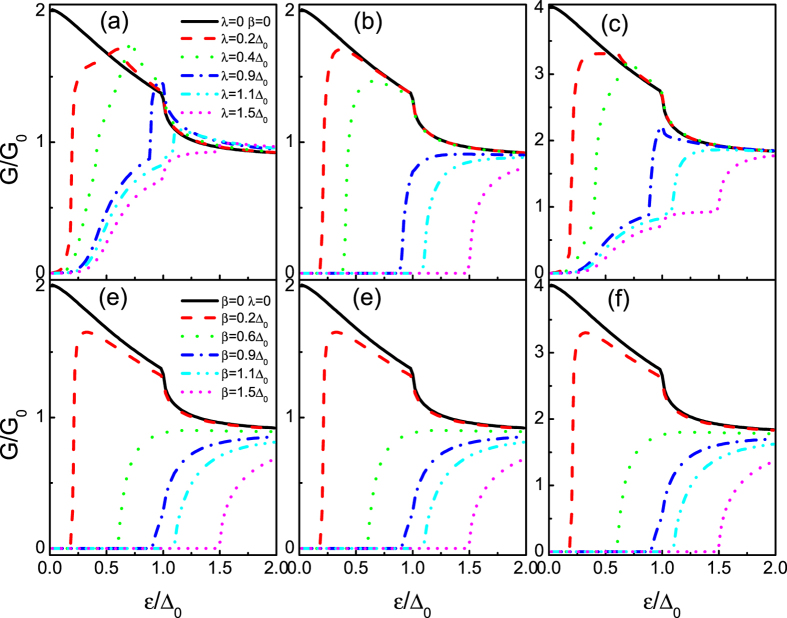
The tunneling conductance for the superconducting heterojunction as a function of the incident energy *ε* for the different *λ* (**a–c**) and *β* (**d–f**). From the left panel to the right panel, *G*′ = *G*_−_, *G*_+_, *G*. The parameters used in the calculation are *l* = 0, *V*_0_ = 0, *V*_*S*_ = 100Δ_0_, *E*_*F*_ = 0, and Δ_0_ = 1. The other parameters are shown in the figure.

We now turn to the case of *E*_*F*_ = 0, i.e., the tunneling conductance completely stems from the SAR. In Fig. 5(a–c), we plot the tunneling conductances as a function of the incident energy *ε* for the different *λ*. The tunneling conductances VS *ε* for different *β* are shown in Fig. 5(d–f). It can be seen clearly that both of them exhibit the similar feature as the case of *E*_*F*_ = 100Δ_0_. Although there is the similar tendency of the tunneling conductance (with the SOI) for the two cases (only the DSOI or only the RSOI), some differences can be also found. In the case of the RSOI, the subgap conductance *G*_−_ always exhibits two inflection points. Indeed, in the energy regime 0 ≤ *ε* < *λ*, there is only one species of spin involved in the SAR process leading to a sharp suppression on the subgap conductance. Beyond the critical point *ε* = *λ*, two species of spin are involved in the SAR process leading to an enhancement on the subgap conductance. It will reach its maximum at a certain incident energy *ε*″ which is the RSOI sensitive. It is important to point that the maximum is larger than the value for the case of without the RSOI. Furthermore, the subgap conductance *G*_+_, in the energy regime 0 ≤ *ε* < *λ*, equates to zero. This nontrivial zero conductance arises because the chirality + electron in the energy gap is just an evanescent transmission wave, as shown in Fig. 3(b), thereby leading to a perfect reflection (a zero conductance). In the case of the DSOI, we can find in Fig. 5(d–f) that the subgap tunneling conductance has the similar feature as the case of *G*_↓_ (only with RSOI). It also can be elucidated in the same manner. Moreover, in contrast to the RAR case (*E*_*F*_ = 100Δ_0_) where it increases with *ε*, the subgap tunneling conductance *G* drops grossly with *ε* (beyond *λ* and *β*) for the DSOI case. These remarkable features show that the subgap tunneling conductance can be tuned largely by the SOI, which suggest that the SOI can be regarded as a key point for diagnosing the SAR.

**Figure 6:**
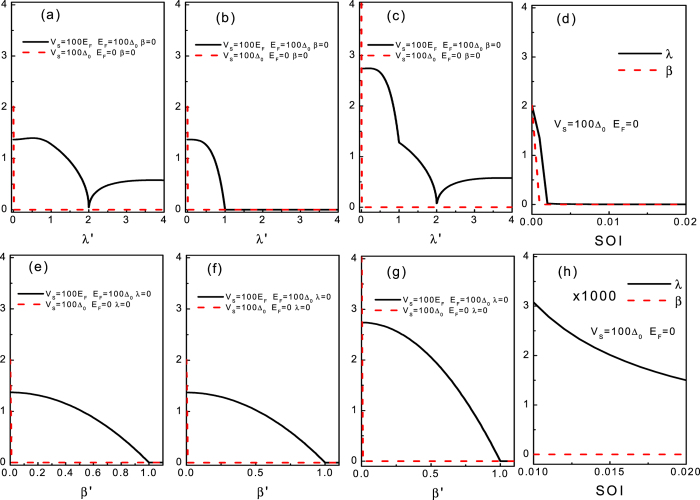
Plot of zero-bias tunneling conductance as a function of the DSOI and the RSOI. The first, second, and third panels correspond to *G*_−_, *G*_+_, *G*, respectively. (**d**) Denotes the comparing results between the DSOI and the RSOI at *E*_*F*_ = 0. (**h**) Denotes the comparing results between the DSOI and the RSOI at *E*_*F*_ = 100Δ_0_. *λ*′ denotes *λ*/*E*_*F*_ and *λ*/Δ_0_ for the case of *E*_*F*_ = 100Δ_0_ and *E*_*F*_ = 0, respectively. *β*′ denotes *β*/*E*_*F*_ and *β*/Δ_0_ for the case of *E*_*F*_ = 100Δ_0_ and *E*_*F*_ = 0, respectively. The other parameters are shown in the figure.

In order to reveal the detailed dependence of the tunneling conductance on the SOI, we will investigate the angular averaged tunneling conductance as a function of the SOI at a zero bias voltage (*ε* = 0). A plot of the zero-bias tunneling conductance as a function of the SOI for the different situation *E*_*F*_ = 100Δ_0_ and *E*_*F*_ = 0, shown in Fig. 6, confirms the analysis above. We first focus on the case of *E*_*F*_ = 100Δ_0_. From Fig. 6, we find that *G*_−_(*ε* = 0) shows a maximal value at *λ* ~ 0.6*E*_*F*_ and then vanishes completely at *λ* = 2*E*_*F*_. Beyond the threshold, it increases again. The complete suppression of the tunneling conductance ascribes to the interference between the chirality − propagating wave and the chirality + evanescent wave. At *λ* = 2*E*_*F*_, the module of the propagating wave and the module of the evanescent wave equate to each other, yields a destructive interference, thereby a perfect reflection. That is to say, the evanescent wave can lead to both a phase and amplitude balance causing an ideal and perfect reflection resonance condition at *λ* = 2*E*_*F*_ and *ε* = 0. However, in the case of *G*_+_(*ε* = 0), it monotonically decreases as a function of *λ* until reaches zero (beyond *λ* = *E*_*F*_, it always equates to zero). Similar to the subgap tunneling conductance above (Fig. 5), the zero-bias tunneling conductance for the case of the DSOI alone shows the same features as *G*_+_(*ε* = 0) (the RSOI alone). While for *E*_*F*_ = 0, the zero-bias tunneling conductances for the RSOI and the DSOI exhibit the same character at the first glance, i.e., they are sharply destroyed by a negligible *λ* or *β*. However, in contrast to the situation of *G*_+_(*ε* = 0), *G*_−_(*ε* = 0) for the RSOI and the DSOI does exhibit some distinct characters. First, it can be seen clearly from Fig. 6(d) that the attenuation trend of the amplitudes of *G*_−_(*ε* = 0) for the RSOI is slightly slower than that for the DSOI. Second, in contrast to the case of the DSOI (with an absolute zero value beyond the threshold), *G*_−_(*ε* = 0) exhibits a monotonical decrease behavior and shows a non-zero negligible value. Thus those qualitatively different characteristics between the case of *E*_*F*_ = 100Δ_0_ (RAR) and *E*_*F*_ = 0 (SAR) provide a heuristic pathway for distinguishing the SAR by the zero-bias tunneling conductance.

#### Barrier effect

**Figure 7:**
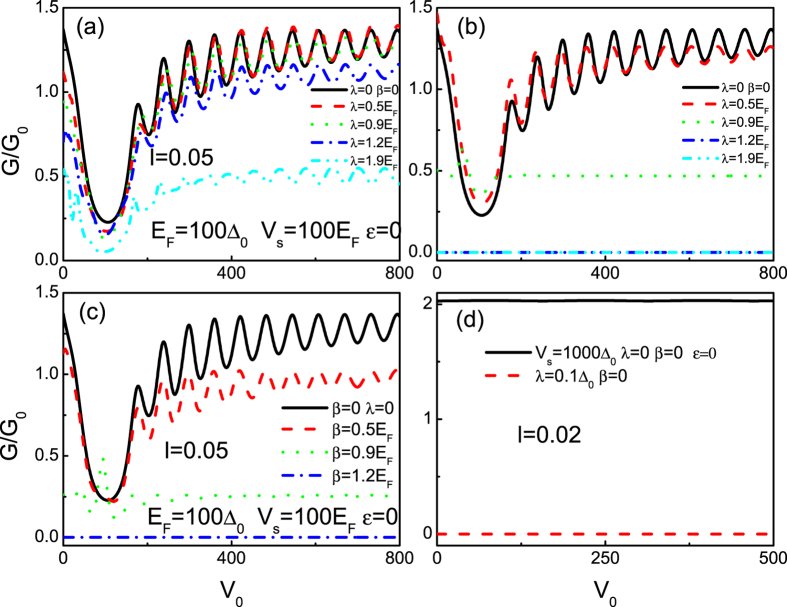
Plot of zero-bias tunneling conductance as a function of the barrier height. (**a,b**) correspond to *G*_−_ and *G*_+_ (for *E*_*F*_ = 100Δ_0_) at the different RSOI, respectively. (**c**) denotes the counterpart results for *E*_*F*_ = 100Δ_0_ at the different DSOI. Due to spin degeneracy, we only show the results of *G*_−_. (**d**) denotes the comparing results at *E*_*F*_ = 0. Due to the similarity, we only show the results of *G*_−_ (for *E*_*F*_ = 100Δ_0_) at a zero and the small value of the RSOI. The other parameters are shown in the figure.

To gain a general overview over the basic tunneling properties through the system, we also investigate the effect of barrier between the SOI lead and the superconducting lead. Figure 7 shows the barrier height dependence of chirality-resolved zero-bias tunneling conductance at different SOI for a fixed barrier width. The parameters are shown in the figure. Note that the zero-bias tunneling conductance for *E*_*F*_ = 100Δ_0_ (RAR) oscillates with the barrier height *V*_0_, as shown in Fig. 7(a–c). It is worth pointing out that, in contrast to the monotonic decay effect in conventional barrier case, the oscillation effect origins from the Fabry-Perot type interferences of relativistic fermions (electron and hole). Comparing them with the case of without the SOI (*λ* = *β* = 0), we find that the oscillation periods and amplitudes are decrease with increasing the SOI. In particular, when the strength of the SOI (*λ* or *β*) exceeds the Fermi energy *E*_*F*_, *G*_+_(*ε* = 0) (the RSOI alone) and *G*_−(+)_(*ε* = 0) (the DSOI alone) equate to zero without the oscillation features. Besides the oscillation, it is also noted from Fig. 7(a–c) that a broad dip turns up around V_0_ = *E*_*F*_ which means that the chirality-resolved zero-bias tunneling conductance can be largely controlled by the barrier height. Moreover, the zero-bias tunneling conductance are asymmetric with respect to the critical point V_0_ = *E*_*F*_. From the theorical point of view, the asymmetric effect can be elucidated by the type of quasiparticles involved in the tunneling process. Specifically, for the case of V_0_ < *E*_*F*_, it can be regarded as a classical motion, at least from the point of view of the transmission. While, it must be a Klein tunneling process in the case of V_0_ > *E*_*F*_. Meanwhile, the same oscillating features below and above the point V_0_ = *E*_*F*_ stem from the distinct quasiparticle types, i.e., electron quasiparticles (classical motion) and hole quasiparticles (Klein tunneling). However, for the case of *E*_*F*_ = 0 (SAR), the situation becomes much different, shown in Fig. 7(d). The oscillation and dip features all disappear no matter which kind of the SOI exits (the RSOI or the DSOI). Furthermore, the zero-bias tunneling conductances for the RSOI and the DSOI exhibit the similar characters. In fact, such striking features can be found a good agreement with the analysis in the above (as shown in Fig. 6) and can be elucidated in a similar way. Actually, the Fabry-Perot type interferences in the barrier region also can be tuned by the length of the barrier *l* (similar to the barrier height V_0_), which is not shown here. Based on the above characteristics of the spin-resolved zero-bias tunneling conductance, we recognize that both the barrier and the SOI can be used as the design variables to tune device properties and optimize circuit performance in future integrated circuits based on graphene materials.

#### Both the RSOI and the DSOI are finite

**Figure 8:**
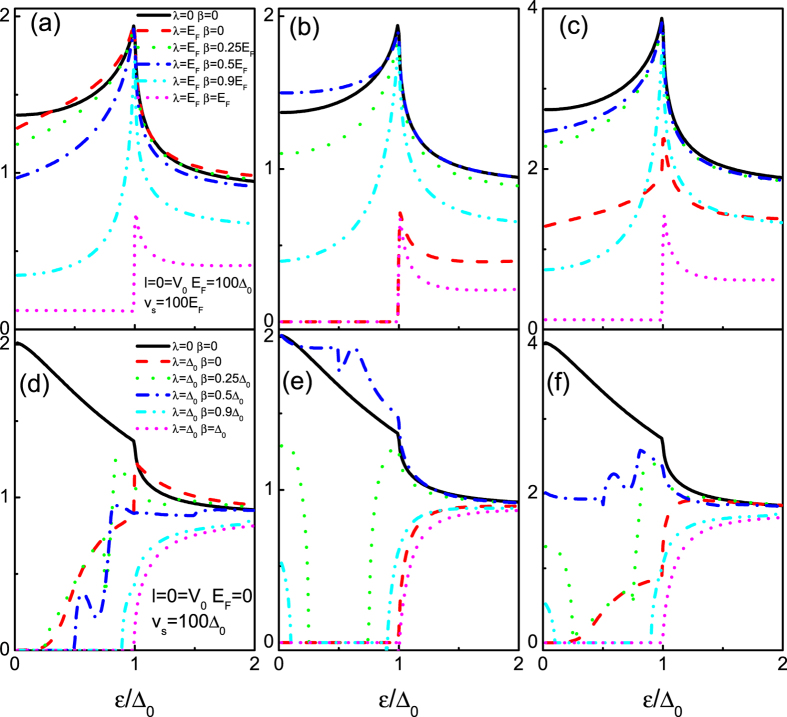
The tunneling conductance for the superconducting heterojunction as a function of incident energy *ε* for *E*_*F*_ = 100Δ_0_ (**a–c**) and *E*_*F*_ = 0 (**d–f**). From the left panel to the right panel, *G*′ = *G*_−_, *G*_+_, *G*. The other parameters are shown in the figure.

Finally, we discuss the intermediate situation that both the RSOI and the DSOI are finite, as shown in Fig. 8. Since the combined effect of the RSOI and the DSOI breaks the particle hole symmetry (Figs 2 and 3), it is expected that the interplays between the RSOI and the DSOI can significantly affect the AR (the RAR and the SAR) in the present superconducting heterojunction. Clearly, in the absence of the SOI, the tunneling conductance reaches its well known features (the subgap conductance increases from 4/3 to twice the ballistic value in the case of the RAR, but it drops from twice to 4/3 in the case of the SAR) as a function of the incident energy, as it should^30^. When both the SOI are finite, the tunneling conductances exhibit more intriguing structure. For the case of *E*_*F*_ = 100Δ_0_ (RAR), in contrast to the chirality − band (*G*_−_ monotonically decays with the DSOI), the chirality + conductance band turns up with increasing the DSOI, thereby enhances the subgap conductance. The amazing thing occurs at the balanced case *λ* = 2*β*, i.e., the enhanced subgap conductance *G*_+_ exceed the limit case (without the SOI). It is also notable that the maximum of *G*_+_ also is achieved at this balance point. If the DSOI becomes large enough (*β* ≥ *λ*), the chirality-resolved conductance bands die away, thereby no RAR can take place leading to a zero subgap conductance. If we set *E*_*F*_ = 0 (SAR), the Mexican-hat-like shape dispersion and the destroy of the particle hole symmetry make the tunneling conductance (Fig. 8(d–f)) exhibit more exotic characteristics as compared to the former case (RAR). Indeed, as shown in Fig. 3(e), the DSOI opens a gap 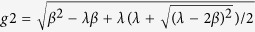 at *k*_*x*_ = 0 separating the chirality − conduction band from the Fermi energy *E*_*F*_ = 0. The gap corresponds to the chirality − evanescent wave transmission, thereby a zero tunneling conductance as a function of the incident energy is found (Fig. 8(d)). Meanwhile, at *k*_*x*_ = 0 a gap 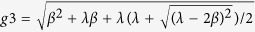 opens between the chirality + conduction band and the Fermi energy *E*_*F*_ = 0. At the energy interval [*g*2, *g*3], one propagating spin band turns up leading to a nonzero tunneling conductance. At the balanced case, three propagating chirality-resolved bands are involved in the tunneling process and the interference between different dispersion relations (the linear and parabolic dispersion) leads to a resonance peak behavior. If the incident energy becomes large enough (beyond *g*3), two chirality-resolved bands are involved in the SAR process, and then results in a large enhancement of the tunneling conductance. The chirality + tunneling conductance curves are shown in Fig. 8(e). For the small DSOI, a zero tunneling conductance gap as a function of the incident energy *ε* is found. The gap lies in the interval of [*g*1, *g*3], corresponding to the gap between the chirality + conductance band and the chirality + valance band. Moreover, the nonzero tunneling conductances below and above the gap show an asymmetry feature. This qualitative difference arises from the fact that the tunneling conductances of the two energy regimes origin from the different kind of AR, where it is valance RAR for the regime [0, *g*1] and SAR for the regime [*g*3, Δ_0_]. In particular, at the balanced case, the subgap tunneling conductance is enhanced largely (exceeding the value of without of the SOI). The behavior is similar to the case of *E*_*F*_ = 100Δ_0_ (RAR) and can be attributable to the reformed line dispersion at the balanced case. For the DSOI large enough (beyond *β* = *λ*), the Mexican-hat-like shape dispersion disappears and the chirality + conductance band shows a gap bigger than the superconducting gap. As a result, the SAR process shuts down and results in a zero tunneling conductance. These phenomena mean that the diagnosing of the SAR can be readily realized in this geometry.

**Figure 9:**
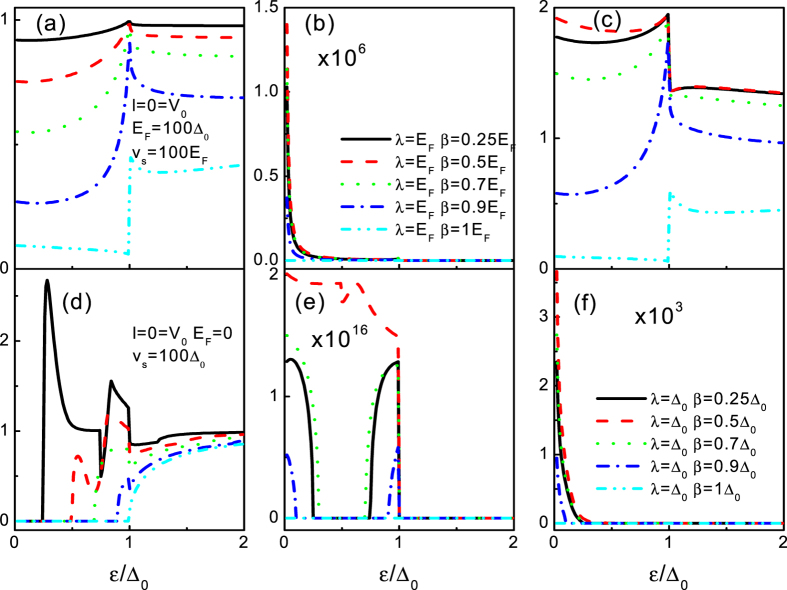
Normalized tunneling conductance *G*_*T*_ = *G*′/*G*′(*λ* = 1, *β* = 0) for the superconducting heterojunction as a function of incident energy *ε* for *E*_*F*_ = 100Δ_0_ (**a–c**) and *E*_*F*_ = 0 (**d–f**). The results correspond to Fig. 8.

To further reveal the effect of the interactions between the RSOI and the DSOI on the AR (the RAR and the SAR), we calculate the normalized tunneling conductance *G*_*T*_ = *G*′/*G*′(*λ* = 1, *β* = 0) in Fig. 9. For *E*_*F*_ = 100Δ_0_, unlike *G*_−_ (always reduced by the DSOI), *G*_+_ shows six orders of magnitude enhancement (the maximum appears at the balanced case). Therefore, the tunneling conductance *G*_*T*_ = *G*/*G*(*λ* = 1, *β* = 0) yields an enhancement also and the maximum (at the balanced case) nearly reaches twice larger. For *E*_*F*_ = 0, both *G*_−_ and *G*_+_ can be enhanced by the DSOI. In contrast to a slightly enhancement for *G*_−_, a giant enhancement can be found with respect to *G*_+_, thereby tunneling conductance *G*_*T*_ = *G*/*G*(*λ* = 1, *β* = 0) shows three orders of magnitude enhancement. These features indicate that the SAR is more sensitive to the SOI than the RAR. The reason is that the energy dispersion is reformed largely by the SOI around the Dirac point. For an electron far away from the Dirac point (the RAR case), the effect of the SOI on the RAR would be much less notable than the SAR case.

**Figure 10:**
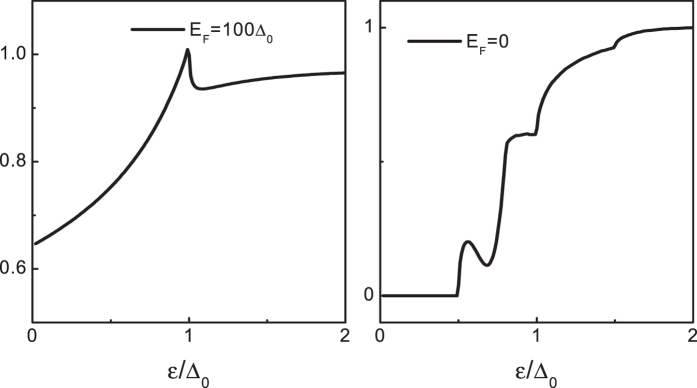
Normalized tunneling conductance *G*_*T*_ = *G*_−_/*G*_+_ for the superconducting heterojunction with *λ* = 2*β* for *E*_*F*_ = 100Δ_0_ (**a**) and *E*_*F*_ = 0 (**b**).

A few remarks are due before we end this study. Of particular interest is the nontrivial case with *λ* = 2*β*. At this balanced case, the chirality + conductance band and the chirality + valance band become linear and combine to form a Dirac cone. While the other pair of chirality-split bands are still parabolic. In this situation, it gives us a unique possibility to test what’s the favorite of the AR (the RAR and the SAR) in graphene, i.e., the linear dispersion incident overwhelms the parabolic dispersion incident, or vice versa. This situation is absent in a pristine graphene and the results are shown in Fig. 10. Clearly, a linear dispersion incident is desired for both the SAR and the RAR. By further comparison, we can draw a conclusion that, in contrast to the RAR where a parabolic dispersion incident and a linear dispersion incident can be comparable with each other, the SAR has a crazy obsession on the linear dispersion. The magical thing is that the RAR indeed is first discovered in the conventional materials with a parabolic dispersion, while the SAR is a unique feature only occurs in the Dirac materials^30^,^31^,^32^,^33^,^50^.

From the experimental point of view, hybrid graphene-based superconducting heterojunctions have attracted much attention since the SAR was revealed theoretically in 2006 by Beenakker. So far, all reports on graphene-based superconducting heterojunctions have been successfully realized in the horizontal tunneling junctions^20^,^21^,^22^,^23^,^51^,^52^ and the vertically stacking tunneling junctions^24^,^53^. It is well known that the potential fluctuation is a common obstacle to reach the case that the SAR dominates and makes the detection of the SAR impossible experimentally in a single-layer graphene. However, here the SOI may make the energy spectrum produce a gap which would suppress the contribution of the RAR (stemming from the potential fluctuation), thereby a nearly pure specular Andreev tunneling conductance yields. Physically, the effect of the SOI is effectively equivalent to dislodging the source of the potential fluctuation. We thus deem that the expected results of the present experimental set-up would be available in the actual experiments in the near future.

## Conclusion

In summary, we have investigated the chirality-resolved transport properties of a superconducting heterojunction in the presence of both the RSOI and the DSOI. We have considered the different experimental limit conditions reported in the literature through the Dirac-Bogoliubov–de Gennes (DBdG) equation simulations: (i) the RAR dominates which is induced by a high Fermi energy and; (ii) a zero Fermi energy leads to the domination of the SAR.

The comparative results of the tunneling conductance for the two cases in the presence of both the RSOI and the DSOI are presented. It is observed that the tunneling conductance not only can be tuned largely by the RSOI, is also related to the DSOI. Such features can essentially benefit the spin-resolved electron devices based on the graphene materials. Besides the similarity, the qualitative differences with respect to those two cases are also revealed.

In the case (i), the retro-Andreev zero bias tunneling conductance exhibits a distinct dependence with respect to the RSOI and the DSOI. However, it is shown that a similar dependence on the two kinds of the SOI for the case (ii). Moreover, in contrast to the case (i) where the zero-bias tunneling conductance oscillates with the barrier parameters, the oscillation phenomena are completely absent in the case (ii). When the RSOI and the DSOI are finite, three orders of magnitude enhancement of the tunneling conductance is found in the case (ii) while just two times enhancement in the case (i). Furthermore, by analyzing the balanced case, we find that the RAR (the case (i)) is in favor of a parabolic dispersion, but a linear dispersion is highly desired for the SAR (the case (ii)).

Therefore, we can expect that our findings could give a significant reference for designing the graphene-based superconducting device via the SOI. It is also hoped that the proposal presented in this study will encourage experimentalists to diagnose the SAR, considering the experimental set-up with the SOI. With the advancement of experimental technology, the SAR should be distinguished based on the graphene materials in the actual experiments in the near future.

## Additional Information

**How to cite this article**: Yang, Y. *et al*. A proposed experimental diagnosing of specular Andreev reflection using the spin orbit interaction. *Sci. Rep.* **6**, 29279; doi: 10.1038/srep29279 (2016).
